# Magnetic Resonance Imaging of Central Nervous System Manifestations of Type 1 Neurofibromatosis: Pictorial Review and Retrospective Study of Their Frequency in a Cohort of Patients

**DOI:** 10.3390/jcm13113311

**Published:** 2024-06-04

**Authors:** Stefano Di Pietro, Linda Reali, Emanuela Tona, Giuseppe Belfiore, Andrea Domenico Praticò, Martino Ruggieri, Emanuele David, Pietro Valerio Foti, Orazio Giuseppe Santonocito, Antonio Basile, Stefano Palmucci

**Affiliations:** 1 Radiology Unit 1, Department of Medical Surgical Sciences and Advanced Technologies “GF Ingrassia”, University Hospital Policlinico “G. Rodolico-San Marco”, University of Catania, 95123 Catania, Italy; 2 Faculty of Medicine and Surgery, University of Enna “Kore”, 94100 Enna, Italy; 3 Unit of Clinical Pediatrics, Department of Clinical and Experimental Medicine, University of Catania, A.O.U. “Policlinico”, P.O. “G. Rodolico”, via S. Sofia, 78, 95125 Catania, Italy; 4 UOSD “IPTRA”, Department of Medical Surgical Sciences and Advanced Technologies “GF Ingrassia”, University Hospital Policlinico “G. Rodolico-San Marco”, University of Catania, 95123 Catania, Italy

**Keywords:** central nervous system, magnetic resonance imaging, type 1 neurofibromatosis

## Abstract

**Background**: type 1 neurofibromatosis (NF1) is the most common neurocutaneous disorder, and it is an inherited condition that causes a tumour predisposition. Central nervous system (CNS) manifestations are a significant cause of morbidity and mortality in NF1. We provide a pictorial review of neuroradiological features of NF1, with emphasis on magnetic resonance imaging (MRI), and we assess the frequency of those features on a cohort of NF1 patients. **Methods**: we retrospectively evaluated all patients with a diagnosis of NF1 who underwent MRI of the spine and brain in our centre over a period of almost 5 years. A total of 74 patients were enrolled, 28 males and 46 females, with a mean age of 21 ± 12.67 years. The frequency of CNS manifestations encountered in our cohort of NF1 patients was assessed and compared with the data found in other studies published in the literature. **Results**: many of our findings were in line with the literature, and possible interpretations for those that turned out to be different were suggested in the discussion. **Conclusion**: imaging plays a central role in the diagnosis and management of NF1, and the knowledge of CNS manifestations could be critical for its early detection and identification, such as for treatment planning and prognostic implications.

## 1. Introduction

Type 1 neurofibromatosis (NF1) is an autosomal dominant inherited condition that causes a tumour predisposition due to inactivating mutations in NF1, a tumour suppressor gene which encodes the RAS inhibitor neurofibromin involved in the regulation of cell growth and survival. This condition is characterized by an extremely variable clinical presentation and it is the most common neurocutaneous disorder, affecting about one out of three thousand persons, without male or female gender predilection; 50% of NF1 cases are familial while the other 50% are sporadic [1,2].

The involvement of the skin, bone, and nervous system is a constant in NF1, since most patients present “café-au-lait macules”, skinfold freckling, Lisch nodules (iris hamartomas), skeletal dysplasia, neurofibromas, and optic gliomas [3]. 

In 2021, the diagnostic criteria for NF1 were revised to incorporate new clinical and genetical discoveries [4]. The diagnosis is generally made when two or more of the following features presented in Table 1 are present.

If an individual has a parent diagnosed with NF1, the diagnosis requires one or more of the criteria reported in Table 1.

Central nervous system (CNS) pathology is a significant cause of morbidity in NF1, and malignant peripheral nerve sheath tumours (MPNST) are the major cause of mortality in adult patients with NF1 [5].

Other NF1 manifestations include musculoskeletal system, lungs, and gastrointestinal regions. Among these extra-CNS lesions, the most represented in order of frequency include the skeletal and muscular manifestations [6]. 

In the assessment of NF1 disease, imaging plays a central role in the diagnosis and management of patients [7]; a careful evaluation of different organs involved is recommended in order to achieve an accurate “staging” of disease and to develop diagnostic and prognostic implications and therapeutic options.

The aim of this work is to provide a pictorial review of neuroradiological features of NF1, with emphasis on MRI images, detailing the technical aspects and the MRI semiotics, and to assess the frequency of those features on a retrospective cohort study based on a population of patients affected by NF1. In the end, we compared our results with those published in the literature.

## 2. Pictorial Review of Central Nervous System Manifestations of Type 1 Neurofibromatosis

### 2.1. Visual Pathway Lesions

Optic pathway gliomas are the most common intracranial tumours, usually represented by low-grade astrocytoma, and occur in 5–15% of NF1 patients [5,8]. They may involve one or both optic nerves, the chiasma, or any portion of the optic tract [5]. 

A clinical suspicion of optic pathway gliomas should be investigated using brain MRI: the examination should include orbits, with and without intravenous administration of contrast material [7]. Brain and orbit MRI, in fact, is the gold standard neuroimaging examination for the diagnosis of optic pathway gliomas in symptomatic NF1 patients [1].

As reported by Mentzel et al. [5], MRI examinations for optic glioma screening should use T1- and T2-weighted sequences, acquired in the axial and coronal planes; axial and sagittal gadolinium-enhanced T1-weighted images, usually not exceeding a slice thickness of 3 mm, are generally recommended to better investigate optic lesions. The oblique sagittal images have to be angled to the optic nerve. Fat saturation is necessary in post-contrast images, in order to separate the enhancing tumour and the intraorbital fat tissue.

**Figure 1:**
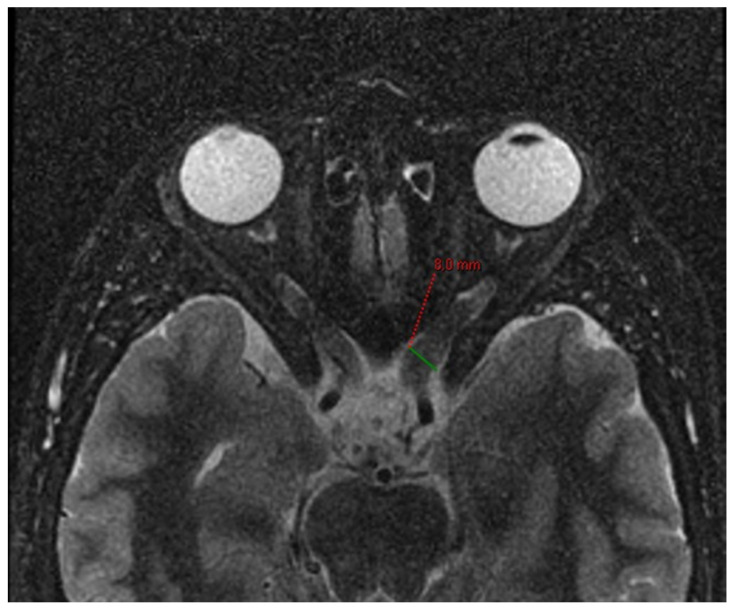
Optic nerve glioma in a patient affected by NF1. Axial T2-weighted FSE with fat suppression. The pre-chiasmatic tract of the left optic nerve has a diameter of approximately 8 mm (green line).

These tumours have a characteristic appearance on T2-weighted images, showing a compact low signal core with a higher intensity circumferential component. Involvement of the chiasma or chiasma tumours are usually demonstrated by diffuse enlargement of the chiasma and optic nerves [8,9]; as reported in a previous paper published by Eid et al., an optic nerve having a diameter greater than 3.9 mm is considered a marker of glioma [10] (Figure 1).

**Figure 2:**
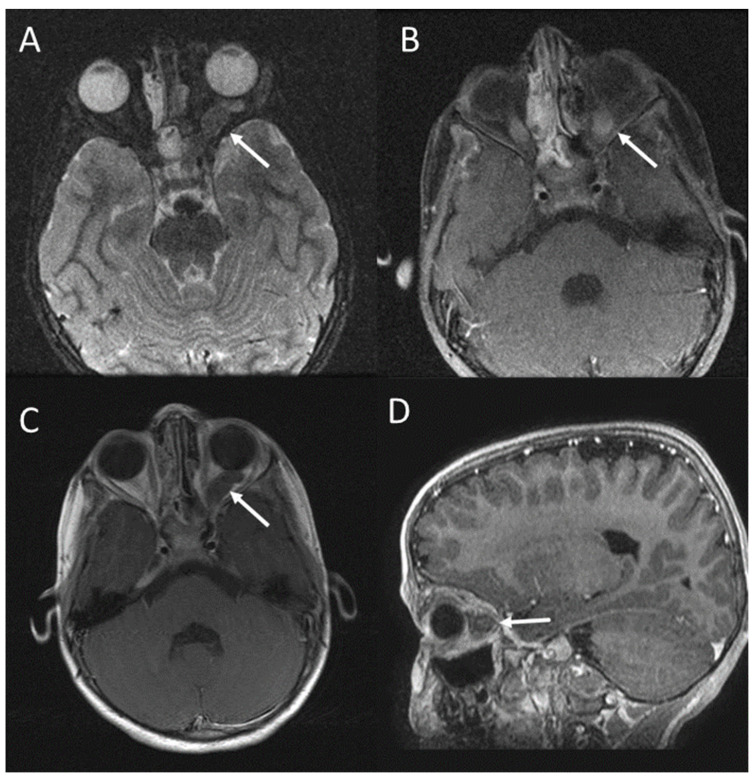
Left optic nerve glioma (arrows) in a patient with NF1. Axial T2-weighted STIR (**A**); axial T1-weighted after gadolinium administration with fat suppression (**B**); axial T1-weighted FSE after gadolinium administration (**C**); sagittal T1-weighted FSPGR after gadolinium administration (**D**) [11].

On T1-weighted images, the optic gliomas and the involved chiasma are isointense [5]. These tumours usually show enhancement after intravenous gadolinium administration [5,8] (Figure 2).

Since the natural course of optic pathway gliomas can vary from aggressive growth to spontaneous regression, follow-up MRI investigations are necessary [5].

### 2.2. Brain Tumours

Patients with NF1 have an increased risk of developing benign and malignant cancers of the central nervous system (CNS). In children, low-grade gliomas are predominant, while high-grade gliomas are more commonly seen in adults with NF1 [8,12].

In children, moreover, gliomas are most commonly localized to the optic pathway and brainstem; however, gliomas could be found also in other areas of CNS, such as the temporal lobes, cerebellum, thalamus, basal ganglia, or spinal cord [12,13]. Among these regions, the cerebellum and the brainstem are the most commonly involved. Since gliomas in NF1 patients are often asymptomatic, many cases are discovered “incidentally” on MRI [5,12]. High-grade gliomas frequently arise in the cerebral hemispheres [12].

MRI is the preferred method of imaging to study brain and spinal cord lesions.

**Figure 3:**
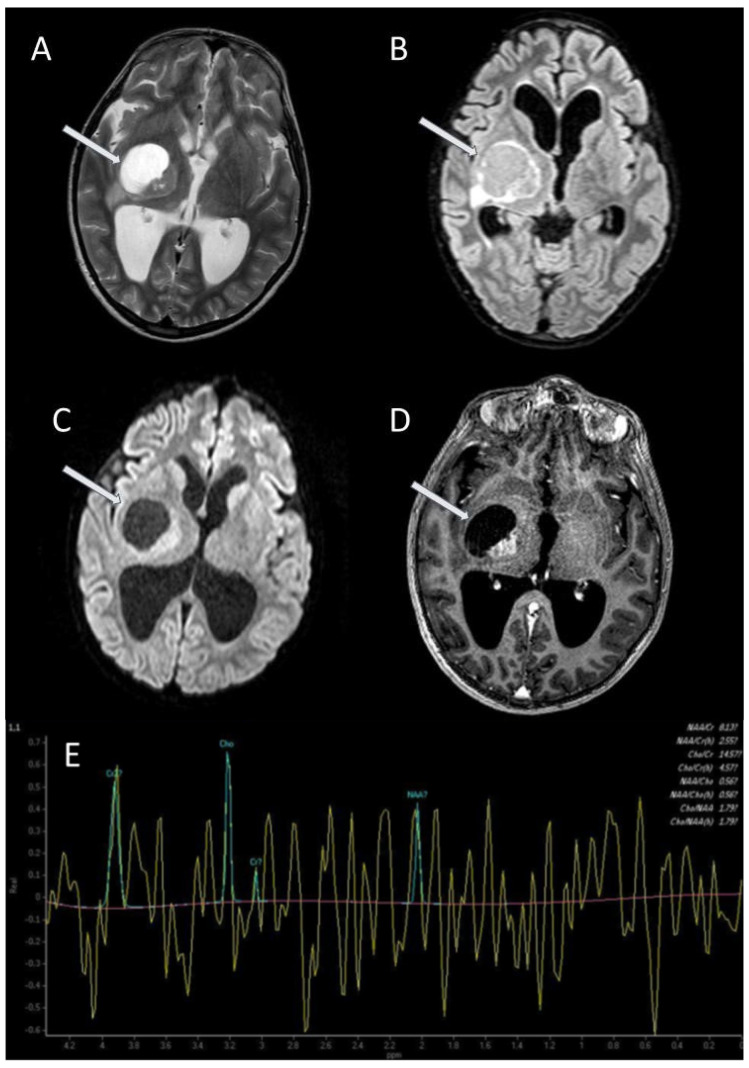
Astrocytoma (arrows) in the right diencephalic and basal ganglia region in a patient with NF1. Axial T2-weighted TSE (**A**); axial 3D FLAIR/SPIR (**B**); axial DWI b1000 (**C**); axial T1-weighted TFE after gadolinium administration (**D**); single-voxel MR spectroscopy (**E**).

There are no radiological differences between gliomas in NF1 and gliomas in non-NF1 cases, appearing as masses isointense or slightly hypointense to normal brain on T1-weighed images, and hyperintense on long TR sequences; in addition, gliomas are usually enhanced after intravenous gadolinium administration (Figure 3) [8].

Since malignant tumours can be discerned from benign lesions only with repeated radiological examinations—or after surgical removal—neuroradiological follow-up investigations are necessary [5].

Currently recommendations for children with known brain tumours suggest to repeat MR examinations 3–6 months after diagnosis. MRI surveillance is not currently recommended unless symptomatic or with an already diagnosed tumour [14].

### 2.3. Brain Abnormalities: UBOs or FASI

**Figure 4:**
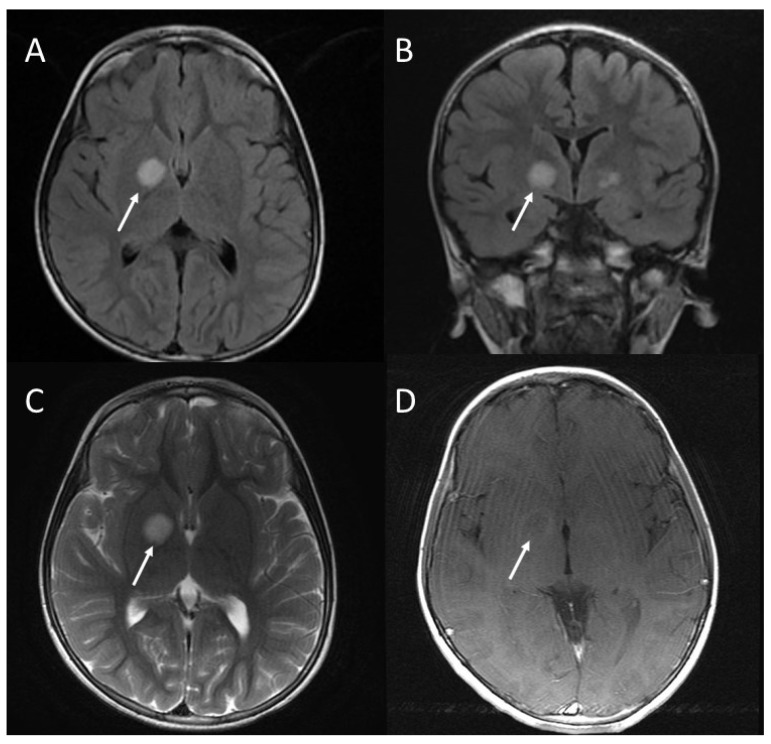
Focal area of signal intensity (FASI) in the right lenticular nucleus (arrows). Axial T2 FLAIR (**A**); coronal T2 FLAIR (**B**); axial T2-weighted FRFSE (**C**); axial T1-weighted FSE after gadolinium administration (**D**) [11].

**Figure 5:**
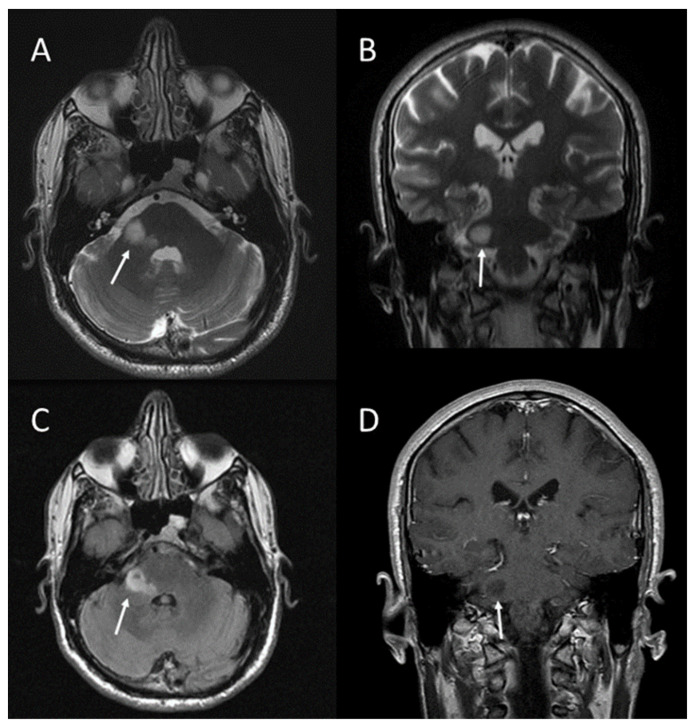
Focal area of signal intensity (FASI) in the right middle cerebellar peduncle (arrows). Axial T2-weighted FRFSE (**A**); coronal T2-weighted FRFSE (**B**); axial T2 FLAIR (**C**); coronal T1-weighted FSE after gadolinium administration (**D**) [11].

UBOs (unidentified bright objects) or, as they have been referred, FASI (focal abnormal signal intensities) are specific lesions which appear as areas of increased signal intensity on T2-weighted MRI sequences. They are not visualized on T1-weighted images, and do not show mass effect or contrast enhancement (Figure 4 and Figure 5). According to the study published by Lopes Ferraz Filho et al. [15], UBOs have been found in a variable percentage, ranging from 43% up to 93% of paediatric NF1 cases; they are rarely seen in patients older than 20 years. The cerebellum, the brainstem, and the basal ganglia are the regions where UBOs are most commonly encountered [16].

Pathologically, according to Di Paolo et al. [17], UBOs correspond to areas characterized by spongiform myelinopathy or vacuolar change of myelin with no inflammatory reaction in the surrounding tissue and no frank demyelination.

Most UBOs regress with age and seem to be benign; however, according to Griffiths et al., some patients have developed tumours in regions with UBOs, suggesting that not all these lesions are benign [18]. Therefore, as suggested by Mentzel et al., MRI follow-up examinations with a contrast agent should be performed in cases with UBOs [5].

Due to high frequency and specificity of UBOs in NF1, these findings may be used as additional criterion for NF1 diagnosis in children, as proposed by some authors [15,16].

**Figure 6:**
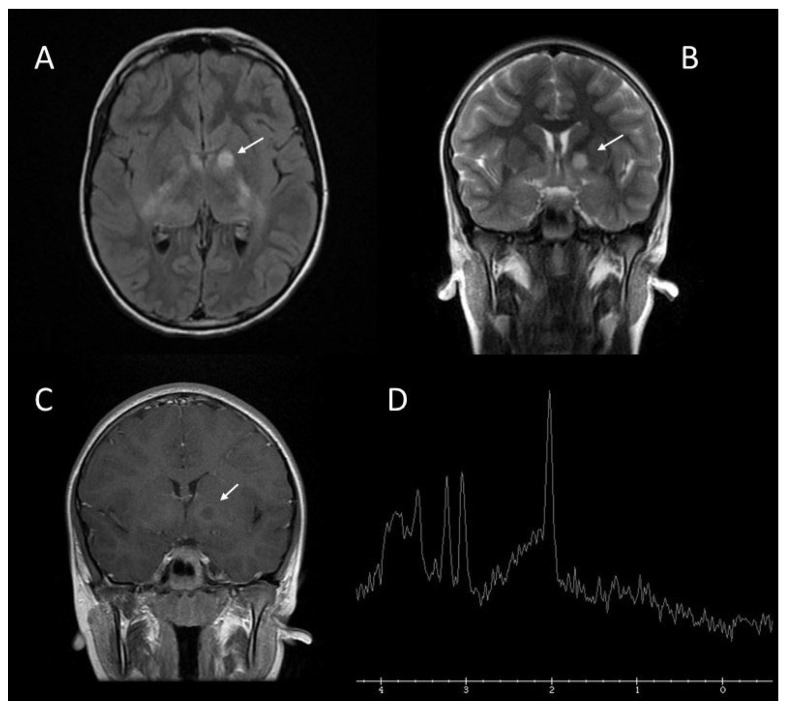
Differential diagnosis between FASI and brain gliomas in the left lenticular nucleus (arrows) using Magnetic Resonance spectroscopy. Axial T2 FLAIR (**A**); coronal T2-weighted FRFSE (**B**); coronal T1-weighted after gadolinium administration (**C**); single-voxel MR spectroscopy revealing a slight decrease in creatine and near normal N-acetylaspartate levels, supporting the diagnosis of FASI (**D**) [11].

In some cases, the differential diagnosis between FASI and brain gliomas can be problematic. Proton magnetic resonance spectroscopy (MRS) can be useful to distinguish between these lesions [19]. A study performed by Gonen et al. [20] reported that MRS reveals distinct metabolic features that differentiate normal, FASI, and tumour regions in the paediatric brain, since MRS shows that tumours exhibit increased choline, decreased creatine (Cho:Cr > 2), and near absence of N-acetylaspartate or lipid signals, while FASI are characterized by elevated choline, reduced creatine (2 > Cho:Cr > 1,3), and near normal N-acetylaspartate levels compared with a normal brain (Figure 3 and Figure 6).

### 2.4. Spinal Tumours

Patients with NF1 can develop benign and malignant spinal tumours. They can be divided into peripheral nerve sheath tumours and intramedullary tumours, originating from the glial cells. The first group includes benign and malignant neurofibromas and benign and malignant schwannomas; the latter group includes medulloblastoma, astrocytoma, meningioma, and ganglioneuroma [21].

A study performed by Thakkar et al. reported different incidence of spinal tumours in asymptomatic and symptomatic NF1 patients, with values of 40% and 96%, respectively [22].

Benign neurofibromas represent the majority of spinal tumours; intramedullary lesions have been rarely observed [23].

**Figure 7:**
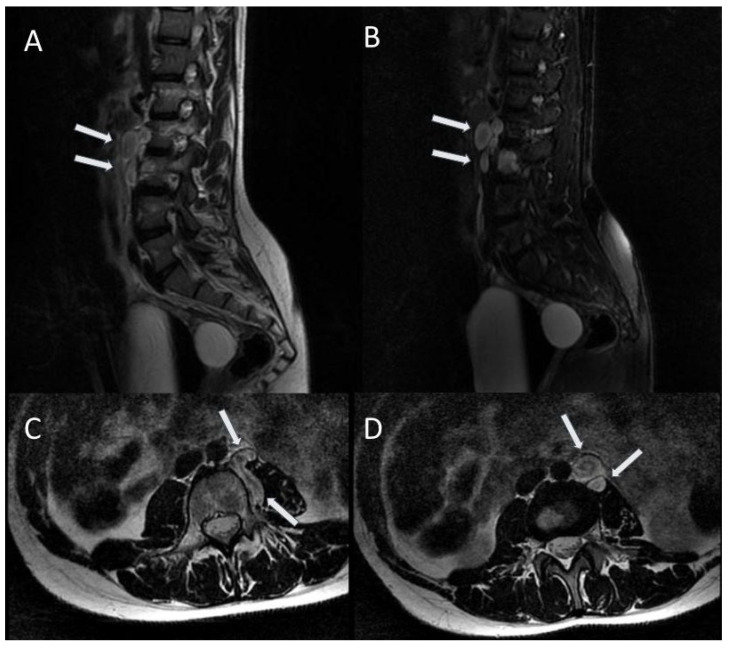
Left prevertebral and paravertebral neurofibromas (arrows) along the L2–L3 vertebrae. Sagittal T2-weighted FRFSE (**A**); sagittal T2-weighted FRFSE with fat suppression (**B**); axial T2-weighted FSE (**C**); axial T2-weighted FSE (**D**) [11].

MRI is useful in the diagnosis and in the follow-up of these lesions; MRI, moreover, can aid in the distinction between benign and malignant nerve sheath tumours, based on the following features suggestive of malignancy: enlarging tumour, tumour size > 5 cm, ill-defined margins, lack of a central hypointense target on T2-weighted images, heterogeneity with central necrosis [23] (Figure 7).

### 2.5. Plexiform Neurofibromas and Sphenoid Dysplasia

Plexiform neurofibromas occur almost exclusively in NF1 and are considered diagnostic in this disease. Plexiform neurofibromas are observed in up to 30% of cases of NF1, most frequently in the craniomaxillofacial region. They contain Schwann cells and perineural fibroblasts, and there is a potential for malignant change into neurofibrosarcoma that is considered an important cause of mortality, occurring in 2% to 16% of cases [5,8,24].

Plexiform neurofibromas arise from major nerve plexus and have the tendency to growth along the nerve fascicles [8], presenting as bulging and deforming masses involving also connective tissue and skin folds that explain why these lesions are often clinically described as “bags of worms” [24].

The first branch of the trigeminal nerve is the most common affected region in the head and neck, often associated with sphenoid dysplasia, other nervous system dysplasias, such as dural ectasia and aqueduct stenosis, and buphthalmos [5,8].

**Figure 8:**
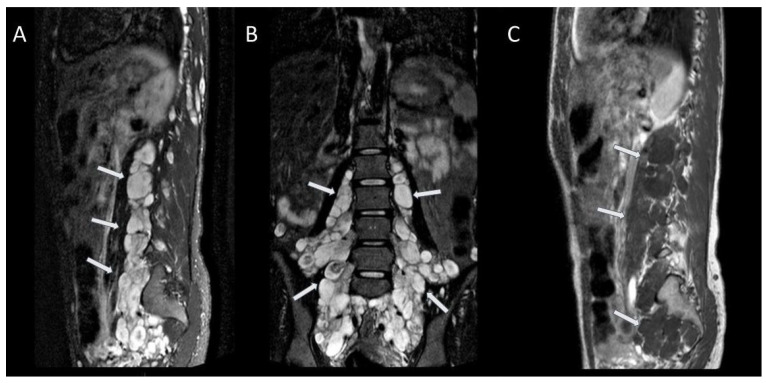
Voluminous plexiform neurofibromas (arrows) in the lumbar, pre-sacral, and pelvic regions. Sagittal STIR (**A**); coronal STIR (**B**); sagittal T1-weighted FSE after gadolinium administration (**C**).

**Figure 9:**
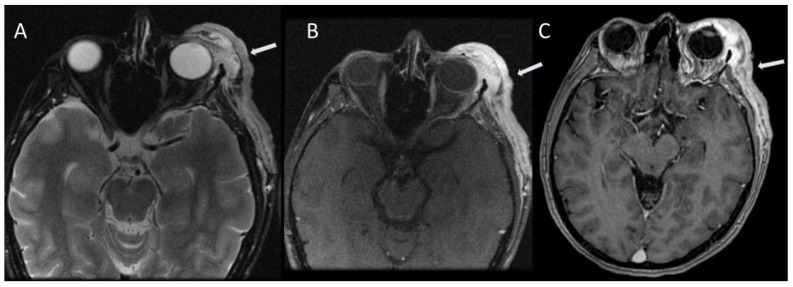
Plexiform neurofibroma (arrows) in the left orbital and temporal region. Axial T2-weighted TSE with fat-suppression (**A**); axial T1-weighted TSE with fat suppression after gadolinium administration (**B**); axial T1-weighted TFE after gadolinium administration (**C**).

Symptomatic plexiform neurofibromas are usually evaluated by imaging studies, such as CT scanning or MRI, which is the modality of choice. Plexiform neurofibromas usually appear as heterogeneous masses with high signal intensity on long TR sequences, often with a central area of low signal, possibly due to fibrous components with surrounding myxoid matrix. On T1-weighted sequences, plexiform neurofibromas show as slightly hyperintense to muscle [8,25]. Variable enhancement after administration of paramagnetic contrast medium has been described in the literature [26] (Figure 8 and Figure 9).

Sphenoid dysplasia frequently manifests as unilateral thinning or a gross defect in the greater or lesser wings of the sphenoid bone, often involving the orbit. It may be clinically asymptomatic in mild cases or it may be associated with loss of vision, progressive proptosis, pulsatile exophthalmos, and facial asymmetry [27].

### 2.6. Other CNS Manifestations of NF1

**Figure 10:**
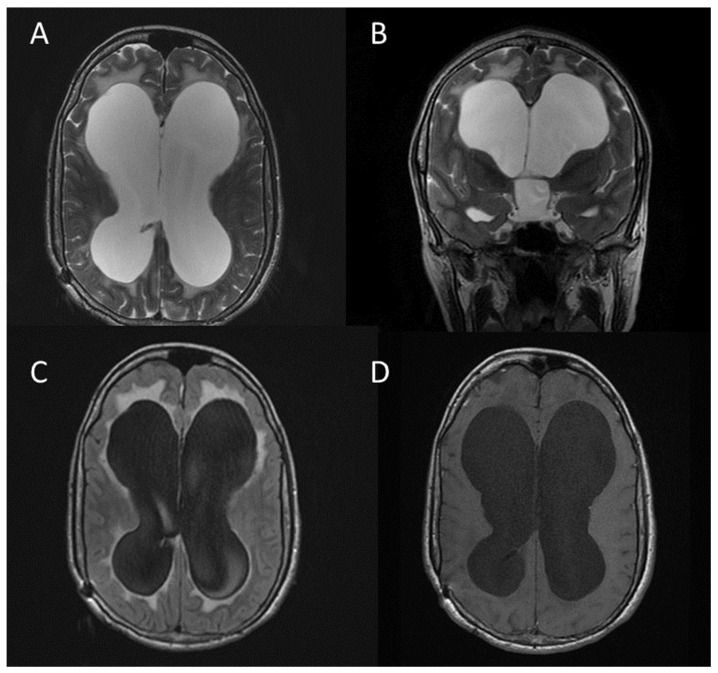
Hydrocephalus in a patient with NF1. Axial T2-weighted FRFSE (**A**); coronal T2-weighted FRFSE (**B**); axial T2 FLAIR (**C**); axial T1-weighted FSE (**D**) [11].

Other CNS abnormalities occur with increased frequency in NF1 [8]:

- Hydrocephalus (usually secondary to aqueduct stenosis) (Figure 10);
- Macrocephaly;
- Meningocoeles;
- Arachnoid cysts;
- Dural ectasia.

Cerebral vasculopathy has been defined as an important but underrecognized complication of neurofibromatosis type 1. Individuals affected by NF1 vasculopathy may be asymptomatic or may present clinical manifestations arising from stenosis or occlusion of the vessels, fusiform aneurysm formation, or arteriovenous fistulae involving the anterior and/or posterior circulation [28].

Moyamoya disease is a rare progressive vaso-occlusive disorder due to a defect in the intima layer, with an estimate incidence of one case per million people per year, involving stenosis of the terminal portions of the internal carotid arteries, their branches, and the main trunk of the anterior cerebral arteries and the middle cerebral arteries [29]. Some authors have found that moyamoya syndrome is quite common, present in 3–5% of children with NF1 with imaging performed for clinical indications [30].

MRI has been adopted for widespread clinical use for assessing cerebral vasculopathy [28], due to the lack of ionizing radiations and to the possibility to detect and study structural variations of the circle of Willis by 3D Time-of-Flight Magnetic Resonance Angiography, without administration of contrast medium.

The key MRI features of the most commonly observed central nervous system manifestations of Type 1 neurofibromatosis and the percentage of patients affected reported in the literature are summarized in Table 2.

## 3. Materials and Methods

### 3.1. Population Study

This study was performed at University Hospital Policlinico “G. Rodolico—San Marco”. Consulting the RIS/PACS system of U.O.C. Radiologia I, we executed a retrospective evaluation of patients with a previous diagnosis of neurofibromatosis type 1 who underwent magnetic resonance imaging (MRI) of the spine and brain from July 2018 to May 2023. A total of 100 MRI examinations were found. Since 17 patients underwent repeated MRI examinations, the first MRI scan for each patient was considered, so a total of 74 MRI examinations performed on 74 patients affected by NF1 were enrolled, and the study population finally included 28 males and 46 females, ranging between 6 and 57 years of age (with a mean age of 21 ± 12.67 years).

The images obtained by MR acquisitions were retrospectively reviewed by two radiologists with proven experience in neuroimaging of NF1.

### 3.2. MR Protocol

All MRI examinations were performed using a 1.5 Tesla MR scanner, using a head neck unit coil for brain and orbit imaging and a high resolution 8-channel phased array coil for spinal cord imaging. All examinations were performed on a Signa HDx MR System (GE Healthcare, Milwaukee, WI, USA), except for five examinations performed on an Achieva 1.5T MRI System (Philips Healthcare, Amsterdam, The Netherlands).

MR imaging was performed with each patient in a supine position. Patients observed no specific diet restrictions or hydration prior to MR examinations. When not contraindicated, intravenous gadolinium contrast agent was administrated. All patients provided written consensus prior to the MR examination.

Our MR study protocol is summarized in Table 3.

### 3.3. Statistical Analysis

MRI findings and medical records were documented using an Excel worksheet (Excel version 2308, build 16, Microsoft Cooperation, Redmond, WA, USA). Descriptive statistics for continuous variables were expressed as arithmetic mean, standard deviation (SD), minimum and maximum (range), and absolute (n) and relative (%) proportions. Categorical variables were expressed as absolute (n) and relative (%) proportions. Data analysis and statistics were performed using MedCalc Statistical Software (MedCalc version 18.2.1, MedCalc Software bvba, Ostend, Belgium).

## 4. Results

### 4.1. Frequency of Visual Pathway Lesions

Fifteen patients (20.3%) with visual pathway lesions were identified and in seven cases (9.4%), we observed the involvement of both optic nerves.

### 4.2. Frequency of Brain Tumours

Four patients (5.4%) with brain tumours were identified. In two (2.7%) cases the tumour was localized in the supratentorial region, while in the remaining two (2.7%) cases the tumour was localized in the infratentorial region.

### 4.3. Frequency of Brain Abnormalities—FASI or UBOs

Forty-three patients (58.1%) with focal abnormal signal intensities (FASI) were identified. In all cases, the basal ganglia, the cerebellum, or the brainstem were involved.

### 4.4. Frequency of Spinal Tumours

Twenty-five patients (33,8%) with spinal tumours were identified.

In all cases, the identified lesions were compatible with benign neurofibromas.

No intramedullary tumours were identified.

### 4.5. Frequency of Plexiform Neurofibromas

Twelve patients (16.2%) with plexiform neurofibromas were identified.

### 4.6. Frequency of Craniofacial Bone Alterations

Craniofacial bone alterations were identified in three patients (4.1%). In two cases (2.70%), we observed the presence of craniosynostosis; in one case (1.3%), we identified sphenoid dysplasia.

### 4.7. Frequency of Other CNS Abnormalities

During the image analysis of all patients, we identified some other CNS abnormalities in six patients (8.1%), not necessarily NF1-related:

- triventricular hydrocephalus in two patients;
- buphthalmos in one patient;
- cerebrovascular anomalies in one patient;
- choroid plexus xanthogranuloma in one patient;
- Hydromyelia in one patient.

### 4.8. Frequency of Normal MRI Examinations

Nine patients (12.1%) had normal MRI appearance with no evidence of signal abnormalities or other pathology.

**Figure 11:**
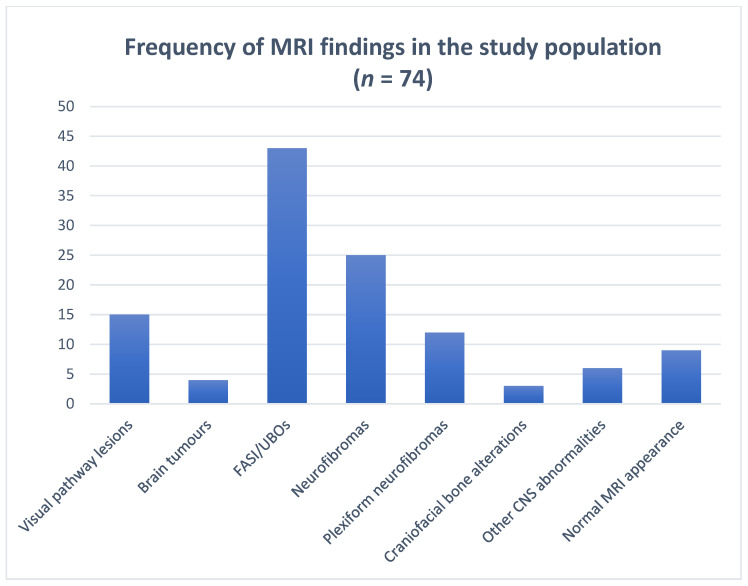
The bar chart shows the frequency of neuroradiological findings encountered on MR imaging of 74 patients affected by NF1. Values are represented as absolute value.

**Figure 12:**
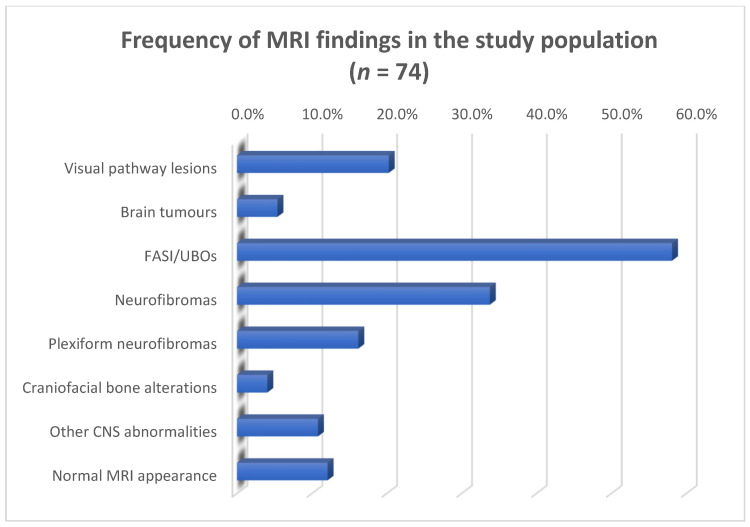
The bar chart shows the frequency of neuroradiological findings encountered on MR imaging of 74 patients affected by NF1. Values are represented as relative proportions (%).

Overall results are summarized in Table 4, Figure 11 and Figure 12.

## 5. Discussion

Visual pathway lesions, generally represented by optic pathway gliomas and usually demonstrated with MRI by diffuse enlargement of the chiasma and optic nerves, are a frequent finding in our sample of patients: 20.3% of patients displayed this finding, although this is a bit higher than the rate of 5% to 15% of cases reported in the literature [5,8].

Bilateral optic pathway gliomas, which are considered pathognomonic for NF1 [10], were found in 9.4% of our cohort of patients.

Brain tumours are usually represented by low-grade gliomas and generally asymptomatic, even if high-grade gliomas can be found in adults with NF1. In our sample, they were found in 5.4% of patients, and this rate is in line with those found in other studies published in the literature [12].

Brain abnormalities such as UBOs or FASI are the most common findings in neuroimaging studies of patients affected by NF1. In the literature, they have been found in a variable percentage of patients, ranging from 43% up to 93% of patients. In our study, FASI have been found in 58.1% of patients, in line with the above-mentioned rates published in the literature. Moreover, also in line with the literature, even in our cohort of patients, the cerebellum, the brainstem, and the basal ganglia were the regions where FASI were most commonly encountered [16].

Spinal tumours can frequently occur in patients with NF1. Benign neurofibromas, arising from the peripheral nerve sheath, are the most common, while intramedullary tumours are rarely found [23]. Except for cutaneous neurofibromas, which are encountered in almost all patients affected by NF1 (99%), subcutaneous or deep neurofibromas are reported to be found in about 15% of patients [31]. In our cohort of patients, neurofibromas have been encountered in 33.8% of patients, a significantly higher rate compared to those reported in the literature. However, the reported frequency of 15% for subcutaneous/deep neurofibromas is based on clinical exams, while the frequency in whole-body MRI is reported to be 2–3x higher [32], so our results can be considered in line with those published in the literature.

Plexiform neurofibromas are considered diagnostic in this disease, since they occur almost exclusively in NF1, and they are observed in about 30% of patients affected by NF1. In our cohort of patients, plexiform neurofibromas have been found in 16.2% of patients, although this is much lower than the above-mentioned rate reported in the literature [5,8,24].

The central role of imaging, in particular, emerges in the early detection and diagnosis of malignant peripheral nerve sheath tumours, which may be the malignant transformation of plexiform neurofibromas. In fact, plexiform neurofibromas are associated with high morbidity, can infiltrate surrounding soft tissues, and can cause compression of the airway or spinal cord and disfigurement. Moreover, NF1 is associated with an 8–13% lifetime risk of developing MPNST, which are aggressive tumours that metastasise widely and have a poor prognosis. Surgical removal is the only treatment option for both manifestations [2,3]. Therefore, the early detection and diagnosis of these tumours could have strong clinical and prognostic implications.

Sphenoid wing dysplasia is considered an almost pathognomonic craniofacial bone anomaly in NF1, and it is reported to occur in about 5% to 12% of cases. Moreover, more than 50% of patients with sphenoid wing defects are found to be affected by NF1 [33]. In our cohort of patients, however, we have identified this distinctive craniofacial bone alteration in only one patient (1.3%).

Furthermore, in two out of seventy-four patients (2.7%), we have identified the presence of craniosynostosis. Although this is not a distinctive craniofacial bone alteration for NF1, some other cases of craniosynostosis in patients affected by NF1 have been reported in the literature [34].

Among the other CNS abnormalities encountered in our cohort of patients, although they are not distinctive for NF1, most of them have an increased incidence in patients affected by NF1.

Hydrocephalus, in fact, has an estimated reported prevalence ranging between 1% and 13% among patients with neurofibromatosis type 1 [35]. In our cohort of patients, we encountered hydrocephalus in two patients (2.7%), therefore our data are in line with those found in the literature.

Buphthalmos, identified in one patient of our study population (1.3%), has been thought to be a possible manifestation of orbitofacial neurofibromatosis, and some cases have been reported in the literature [36].

Even cerebrovascular anomalies, which we encountered just once in our cohort of patients (1.3%), have been described in patients affected by NF1 in other studies found in the literature. In a study conducted in a paediatric population with NF1, 2.5% of children were reported to have cerebrovascular abnormalities [37].

To our knowledge, there are no studies published in the literature that could suggest a correlation between choroid plexus xanthogranuloma and hydromyelia with neurofibromatosis type 1, so they could be considered as incidental findings.

Nine patients (12.1%) had normal MRI examinations. On a study conducted by Ferner et al. [38], 38 patients with NF1 underwent MRI of the brain, and in 10 (26.3%) of them no MRI abnormalities were found. Moreover, on another study performed by Van Es et al. [9], 50 children underwent MRI examination and 16 of them (32%) had normal MRI examination. These results are different from those find in our study, but it should be considered that our methodology of study included MRI of the spine in addition to MRI of the brain.

We are aware that this study has some limitations, since it is limited to a single centre experience, and it has a limited number of enrolled patients. Moreover, because of the retrospective design, the presence of other relevant clinical characteristics or any associated diseases was not assessed. Finally, in our study, five subjects were examined using a different MRI scanner; although the imaging acquisition technique and the MR protocol were almost the same, and the pulse sequences used were as similar as possible, the possibility that different scanners had an impact, however small, on the results of this study cannot be ruled out.

## 6. Conclusions

Imaging plays a central role in the diagnosis and management of NF1. Radiologists should be aware of neuroimaging findings since the early detection and identification of central nervous system manifestations of NF1 could be critical for treatment planning and prognostic implications.

The knowledge of the frequency of the most common features encountered in magnetic resonance imaging of the brain and spine in patients affected by NF1 could be helpful in paying due attention to their research and identification, although incidental findings should not be overlooked.

Further studies may be undertaken with larger samples of patients and a larger number of assessed manifestations of NF1, perhaps including not only the neuroradiological features of this disease, but also those related to musculoskeletal system, lungs, and gastrointestinal tract, to refine understanding of the radiological features of NF1 and their frequency.

## Figures and Tables

**Table 1:** Revised diagnostic criteria for neurofibromatosis type 1 (NF1) in patients who do not have parents diagnosed with NF1.

1	Six or more café-au-lait macules > 5 mm in prepubertal individuals and over 15 mm in post-pubertal individuals
2	Freckling in the axillary or inguinal region
3	Two or more neurofibromas of any type or one plexiform neurofibroma
4	Optic pathway glioma
5	Two or more iris Lisch nodules or two or more choroidal abnormalities
6	A distinctive osseous lesion such as sphenoid dysplasia, anterolateral bowing of the tibia, or pseudarthrosis of a long bone
7	A heterozygous pathogenic NF1 variant with a variant allele fraction of 50% in apparently normal tissue such as white blood cells

**Table 2:** MRI features and frequency of the most common CNS manifestation of NF1.

Finding	% of Patients Affected	MRI Features
Visual pathway lesions (optic pathway gliomas)	5–15%	Enlargement of optic nerves or chiasma (diameter greater than 3.9 mm);
		On T2-weighted images: compact low signal core with higher intensity circumferential component;
		On T1-weighted images: isointense signal, with enhancement after gadolinium administration.
Brain tumours	Variable—based on brain location and grade of tumour	In children, low-grade gliomas are predominant, and the cerebellum and the brainstem are the most involved regions. In adults, high-grade gliomas are more commonly, frequently arising in the cerebral hemispheres;
		On T2-weighted images: hyperintense signal;
		On T1-weighted images: isointense or slightly hypointense signal, with enhancement after gadolinium administration.
Brain abnormalities: UBOs or FASI	43–93%	Areas of increased signal intensity on T2-weighted MRI sequence, not visualized on T1-weighted images and do not show mass effect or contrast enhancement;
		Rarely seen in patients older than 20 years;
		The cerebellum, the brainstem, and the basal ganglia are the regions most involved.
Spinal tumours: peripheral nerve sheath tumours and intramedullary tumours	40–96%	Benign neurofibromas represent the majority of spinal tumours, while intramedullary lesions are rarely observed;
		Neurofibromas usually show hyperintense signal on T2-weighted images with central hypointense target, and hypointense signal on T1-weighted images, with heterogeneous contrast enhancement.
		Features suggestive of malignant nerve sheath tumour: enlarging tumour, tumour size > 5 cm, ill-defined margins, lack of a central hypointense target on T2-weighted images, heterogeneity with central necrosis.
Plexiform neurofibromas	Up to 30%	Observed most frequently in the craniomaxillofacial region;
		On T2-weighted images: heterogeneous masses with high signal intensity, often with a central area of low signal;
		On T1-weighted images: slightly hyperintense to muscle, with variable contrast enhancement.

**Table 3:** MR protocol for brain, spine, and orbit imaging in patients affected by NF1.

	TR (ms)	TE (ms)	Thickness (mm)	Gap (mm)	FOV	Matrix	NEX
**Sagittal 2D FSE T1**	380	12	4	0.5	25	256 × 384	1
**Coronal 2D T2 FRFSE**	6420	101.2	4	0.5	25	224 × 320	4
**Axial 2D T2 FRFSE**	5600	123.5	4	0.5	25	256 × 384	4
**Axial 2D T2 FLAIR**	10,002	136.3	4	0.5	25	224 × 256	1
**Axial 2D DWI b1000**	6850	81.9	4	0.5	25	128 × 128	2
**Axial 2D** **FSE T1**	400	12.4	4	0.5	25	256 × 384	2
**Axial 2D** **T2 GRE**	400	14.4	4	0.5	25	256 × 320	1
**Axial 3D** **b-SSFP**	6.6	2.5	0.8	−0.4	25	256 × 448	4
**Axial 2D FSE T2 FS**	2080	95.9	3	0	16–18	256 × 256	4
**Axial 2D FSE T2**	1880	102.4	3	0	16–18	256 × 256	4
**Axial 2D FSE T1 FS**	360	10.6	3	0	16–18	224 × 256	2
**Axial 3D IR FSPGR T1**	8.5	3.1	2	0	25	224 × 256	2
**3D TOF** **MRA**	23	2.8	1.4	−0.7	16	256 × 320	1
**Sagittal 2D FSE T1**	500	11	3	0.3	36–38	256 × 320	2
**Sagittal 2D FRFSE T2 FS**	5700	107.3	3	0.3	36–38	256 × 320	2

**Table 4:** Frequency of MRI findings in the study population (*n* = 74).

Finding	%
Visual pathway lesions	20.3
With bilateral involvement	9.4
Brain tumours	5.4
Supratentorial region	2.7
Infratentorial region	2.7
FASI/UBOs	58.1
Spinal tumours	33.8
Peripheral nerve sheath tumours	33.8
Intramedullary tumours	0
Plexiform neurofibromas	16.2
Craniofacial bone alterations	4.1
Craniosynostosis	2.7
Sphenoid dysplasia	1.3
Other CNS abnormalities	8.1
Triventricular hydrocephalus	2.7
Buphthalmos	1.3
Cerebrovascular anomalies	1.3
Choroid plexus xanthogranuloma	1.3
Hydromyelia	1.3
Normal MRI examinations	12.1

## Data Availability

Data supporting the findings of this study are available within the paper; some of them are not publicly available due to reasons of sensitivity and are available from the corresponding author upon reasonable request.
